# Characteristics of single-channel electroencephalogram in depression during conversation with noise reduction technology

**DOI:** 10.1371/journal.pone.0266518

**Published:** 2022-04-13

**Authors:** Yasue Mitsukura, Yuuki Tazawa, Risa Nakamura, Brian Sumali, Tsubasa Nakagawa, Satoko Hori, Masaru Mimura, Taishiro Kishimoto

**Affiliations:** 1 School of Integrated Design Engineering, Keio University, Yokohama, Kanagawa, Japan; 2 Department of Neuropsychiatry, Keio University School of Medicine, Shinjuku-ku, Tokyo, Japan; 3 Division of Drug Informatics, Keio University Faculty of Pharmacy, Minato-ku, Tokyo, Japan; Osaka University Graduate School of Medicine, JAPAN

## Abstract

**Background:**

Previous studies have attempted to characterize depression using electroencephalography (EEG), but results have been inconsistent. New noise reduction technology allows EEG acquisition during conversation.

**Methods:**

We recorded EEG from 40 patients with depression as they engaged in conversation using a single-channel EEG device while conducting real-time noise reduction and compared them to those of 40 healthy subjects. Differences in EEG between patients and controls, as well as differences in patients’ depression severity, were examined using the ratio of the power spectrum at each frequency. In addition, the effects of medications were examined in a similar way.

**Results:**

In comparing healthy controls and depression patients, significant power spectrum differences were observed at 3 Hz, 4 Hz, and 10 Hz and higher frequencies. In the patient group, differences in the power spectrum were observed between asymptomatic patients and healthy individuals, and between patients of each respective severity level and healthy individuals. In addition, significant differences were observed at multiple frequencies when comparing patients who did and did not take antidepressants, antipsychotics, and/or benzodiazepines. However, the power spectra still remained significantly different between non-medicated patients and healthy individuals.

**Limitations:**

The small sample size may have caused Type II error. Patients’ demographic characteristics varied. Moreover, most patients were taking various medications, and cannot be compared to the non-medicated control group.

**Conclusion:**

A study with a larger sample size should be conducted to gauge reproducibility, but the methods used in this study could be useful in clinical practice as a biomarker of depression.

## 1 Introduction

The diagnosis of depression and assessment of severity are generally done based on a patient’s subjective answers to a questionnaire about such aspects as mood, concentration, lassitude, psychomotor retardation, insomnia, appetite, etc. However, these aspects are very difficult to assess objectively. Severity assessments with poor objectivity and quantitativeness, which can also be influenced by raters’ experience, are a major barrier in clinical practice and clinical trials.

An electroencephalogram (EEG) records the electrical activity of the brain using scalp electrodes. An EEG is generally used in clinical practice to test for the presence or absence of disturbance of consciousness, identification of epileptic seizures, etc., but many previous studies have also used EEG to evaluate other psychiatric disorders. Among them, the evaluation of depression using EEG has been conducted since the 1930s. Since that time, the relationship between mood and alpha waves has been reported [1]. Furthermore, subsequent studies have reported that left-right differences in α-power values are characteristic of depression [2]. There is also a previous study that clarified that the power spectrum of slow waves is high in patients with depression [3]. In addition, it has been reported that not only slow waves, but also frontal β waves, are strongly emitted in patients with depression [4]. Thus, utilizing EEG in the evaluation of depression may be useful as an objective marker, but the results have been various and inconsistent.

Moreover, a conventional EEG test needs a dedicated EEG measurement room that shields the power supply noise, and it is necessary to have the patient remain silent, as well as have a skilled examiner to operate the EEG test. However, in recent years, technology to remove myoelectricity and other noises in real-time from the EEG has been developed, and techniques for recording EEG during open-eyed behavior have also been developed.

One of the previous studies measured EEG during an open-eyed state and successfully showed the left-right difference between α-wave and slow wave in the frontal lobe [2, 5]. Similarly, another study using open-eyed data suggested that increased θ and α activity in the occipital and parietal regions, and increased β activity in the parietal region, are characteristic of patients with depression [6]. In these studies, a filter that can remove electrical activity caused by blinking was applied, but noise from body movement and the environment was not completely removed. As a result, the extent to which the frequency can be regarded as a disease characteristic is limited, and the same patient may have different results depending on the recording situation.

In this study, we used a new single-channel EEG that can address such problems, and also used software-based noise reduction technology to stabilize the EEG for patients with depression during real-time conversation. By recording EEG during conversation in a normal laboratory environment under open-eyed conditions without noise limitations, it may be possible to determine the EEG features of patients with depression. The contributions of this study are as follows:

1. Firstly, the EEG features for classifying depression patients and healthy volunteers were found.
2. EEG Features that are related to the depression severity were also found
3. Feature analysis with considerations of influence of medications were performed

## 2 Methods

### 2.1 Participants (patients and controls)

This study was approved by the ethics committees of our Keio University School of Medicine Ethics Committee and performed in accordance with the Declaration of Helsinki (No.20190188). Written informed consent was obtained from all participants. The individual in the supplemental material has given written informed consent (as outlined in PLOS consent form) to publish these case details.

Patients who met the following inclusion criteria were recruited: 1) Diagnostic and Statistical Manual of Mental Disorders, Fifth Edition (DSM-5) diagnosis of major depressive disorder (MDD); 2) age ≥20 years.

Exclusion criteria for patients were: 1) persons who have physical or psychiatric disorders that impede the use of EEG; 2) persons who have comorbid psychiatric disorders other than depression; 3) persons who have comorbidities that could interfere with EEG recordings, such as brain tumors, stroke, or epilepsy.

For the comparison, reference data for healthy individuals that were obtained separately from this study were used. Inclusion criteria for healthy individuals were: 1) no history of mental illness; 2) age ≥20 years. It was also required that they do not meet the exclusion criteria for depression patients listed above.

### 2.2 Clinical assessment

Sociodemographic information such as age, sex, illness duration, and treatment history were collected from interviews and/or patients’ charts. Along with the EEG data collection, Hamilton Depression Rating Scale [7] assessments were conducted by skilled raters. Based on HAMD score results, patients with scores of 0–7 were classified as Asymptomatic; 8–13 as Mild; 14–18 as Moderate; 19–22 as Severe; and 23 or more as Profound.

### 2.3 EEG acquisition

Participants were asked to wear a single channel EEG device (NeuroSky Single Channel EEG, Original noise reduction BMD version) (S1 and S2 Figs). The usage of a single-channel EEG device is based on the following reasons:

1. Multichannel EEG setup, although provide more biosignal data, are costly in terms of setup time and comfortability, which during the interview duration may cause ambient stress.
2. There are studies that show the possibility of utilizing FP1 in 10–20 system for diagnosing clinical depression along with the severity. For example, Shen et al. [8] introduced a novel depression diagnosis system using three frontal electrodes while Nakamura and Mitsukura successfully found that there were some frequency bins which had significant difference [9], when compared between depression patients and healthy controls.

For the recording session: First, participants were asked to close their eyes for 30 seconds for the purposes of calibrating the device. The researchers then gathered EEG recordings while the participant engaged in interviews to assess HAMD scores for an approximately 40-minute period. These evaluations were conducted up to three times for each participant. The measurement interval was not fixed, as ideally the next recording was done after each patient’s illness severity had changed to some extent, but an interval of at least one week was left between sessions.

### 2.4 Data preprocessing

For the EEG device used in this study, independent verification has already demonstrated that the device can reliably remove environmental noise and unintended frequencies [10]. Signals acquired from Fp1 using a monopole EEG were passed through a bandpass filter of 1–30 Hz to extract EEG components [11]. However, even if non-target frequencies can be removed, the acquired data come with noise caused by muscle movement or blinking. To remove these noises, a filter created for this purpose was used. This filter acquires the patterns of body movement and blinking in advance, and the threshold value is automatically set according to the situation. We adopted conventional methods as noise reduction [12–14]. The artifact rejection algorithm, in particular, follows the paper [12]. The EEG signal is first processed with FastICA algorithm, then passed to a 50Hz and 60Hz stopband filter to remove power line noise. Soft-thresholding noise removal is additionally applied to prepare for artifact removal. Discrete Wavelet Transform (DWT) is computed afterwards. The second level of DWT Detailed coefficients approximate the 40Hz signal corresponding to the muscular artifacts while the fourth level of approximate coefficients results in a low frequency signal of around 8Hz which corresponds to eye blink artifact. This process is carried out for each of the channels of the FastICA. This procedure reduces computation costs and removes blinks, body movements, and electrical noise in real time.

In order to account for individual differences in EEG amplitude, normalization was performed with an average of 0 and a dispersion of 1 for the filtered signal. Subsequently, a fast Fourier transform was performed to calculate the power spectrum. Also, in order to clarify the difference between healthy individuals and each patient group with depression of different severity, the average power spectrum of the comparison target group was set as 1; i.e., a relative power spectrum value was used.

The data size for each individual was approximately 2400 sec (40 min). The sampling interval of the EEG device was set to 512 Hz. Therefore, the amount of data for each individual was 1,228,800 samples (2400*512). Each individual’s EEG data were translated to the frequency domain by Fourier transform per second.

The features utilized in this study is the individual EEG frequencies from 1 to 30 Hz. Conventional studies [15] have focused on EEG bands which spans into multiple frequencies and therefore are less specific to the types of disease, as many diseases might affect similar frequency ranges. Therefore, the individual frequencies are chosen as measures that represent more detailed measures of depression. The details of the feature extraction can be found on Table 1.

**Table 1:** Comparison of conventional studies and this study, with respect to the utilized features.

Authors	Feature Extraction	Subject	Window Size	Features
Stewart and Allen [2]	Fast Fourier Transform (absolute power)	67 depression 60 healthy	2 seconds	Five frequency bands: delta (1−4 Hz), theta (4−8 Hz), alpha (8−12 Hz), beta (12−30 Hz), and gamma (30−50 Hz) 64-channel setup
Roh et al. [5]	Fast Fourier Transform	111 early depression 526 healthy	4.096 seconds, 50% overlap	Absolute theta (4–7.5 Hz), alpha (7.5–14 Hz), and beta (14–20 Hz) power; each frequency band; grand average of power spectra
Grin-Yatsenko et al. [6]	Current-Source Density (CSD) and Fourier-transform	54 subjects (before-after study)	60 seconds, 1.5seconds overlap	Asymmetry score from total alpha power
This study (Proposed method)	Fast Fourier Transform	40 depression 40 healthy	Whole HAMD session (approx. 40 minutes)	Individual Frequencies from 1 to 30Hz (FP1)

Although gamma EEG band might contain information related to clinical depression, several studies have found that Alzheimer’s [16, 17] and other conditions [18, 19] might also be linked to it. As the objective of this study is to build a foundation on diagnosing depression, gamma band is not included in the analysis to prevent the detecting features unrelated with depression.

### 2.5 Statistical analysis

First, all acquired data were divided into two groups: patients with depression and healthy controls. Descriptive statistics were used to describe the study participants. Distributions of all variables were inspected using histograms, q-q plots, and Shapiro-Wilks tests before conducting statistical analyses. Statistical significance was set at two-tailed p < 0.05, and we used false discovery rate (FDR) to control for multiple comparisons.

Demographic variables for patients with depression and healthy individuals were compared by two-sample t test and/or chi-square test.

For the EEG comparisons, patients’ EEG power spectra are expressed as a ratio when the average of the power spectra of healthy individuals is 1, as mentioned in section 2.4 above. The EEG power spectra for each frequency from the patient group and control group were compared using the t test. First, we compared the EEG of healthy persons and patients with depression as a whole. As mentioned in section 2.3 above, each patient had up to three EEG tests, but in cases where multiple recordings were available, the EEG done at the time of highest symptom severity was used. Next, as shown in section 2.2 above, the patients were classified according to severity of depression and the EEG of each severity group were compared with those of healthy individuals. In addition, in order to examine the effects of any prescribed drugs, patients were split into subgroups based on whether they did or did not take antidepressants, benzodiazapines, or antipsychotics, and were compared with the EEG of healthy individuals. The subgroups of medicated and non-medicated patients were also compared.

## 3 Results

### 3.1 Demographic and clinical information

Sixty-one data sets from a total of 40 patients and 50 datasets from a total of 40 healthy individuals were used in the analysis. Demographic and clinical characteristics are presented in Table 2.

**Table 2:** Demographic characteristics of patients and healthy controls.

	Depression patients (n = 40)	Healthy controls (n = 40)	p-value
Age, in years, Mean(SD)	48.0 (13.5)	40.8 (13.8)	0.62
Female, n(%)	21 (61.8)	20 (50.0)	0.35
Educational years, Mean (SD)	15.8 (6.8)	17.0 (8.0)	0.41
Years with illness, Mean (SD)	6.19 (19.0)	-	-
Use of any antidepressants, n(%)	35 (87.5)	-	
Use of any antipsychotics, n(%)	15 (37.5)	-	
Use of any benzodiazepines, n(%)	27 (67.5)	-	
Use of any antiepileptics, n(%)	3 (7.5)	-	
HAMD17 (SD)*	14.7 (8.7)	-	

*For patients who had multiple evaluations, the highest HAMD score was used.

### 3.2 Comparison of healthy individuals and patients with depression

**Fig 1:**
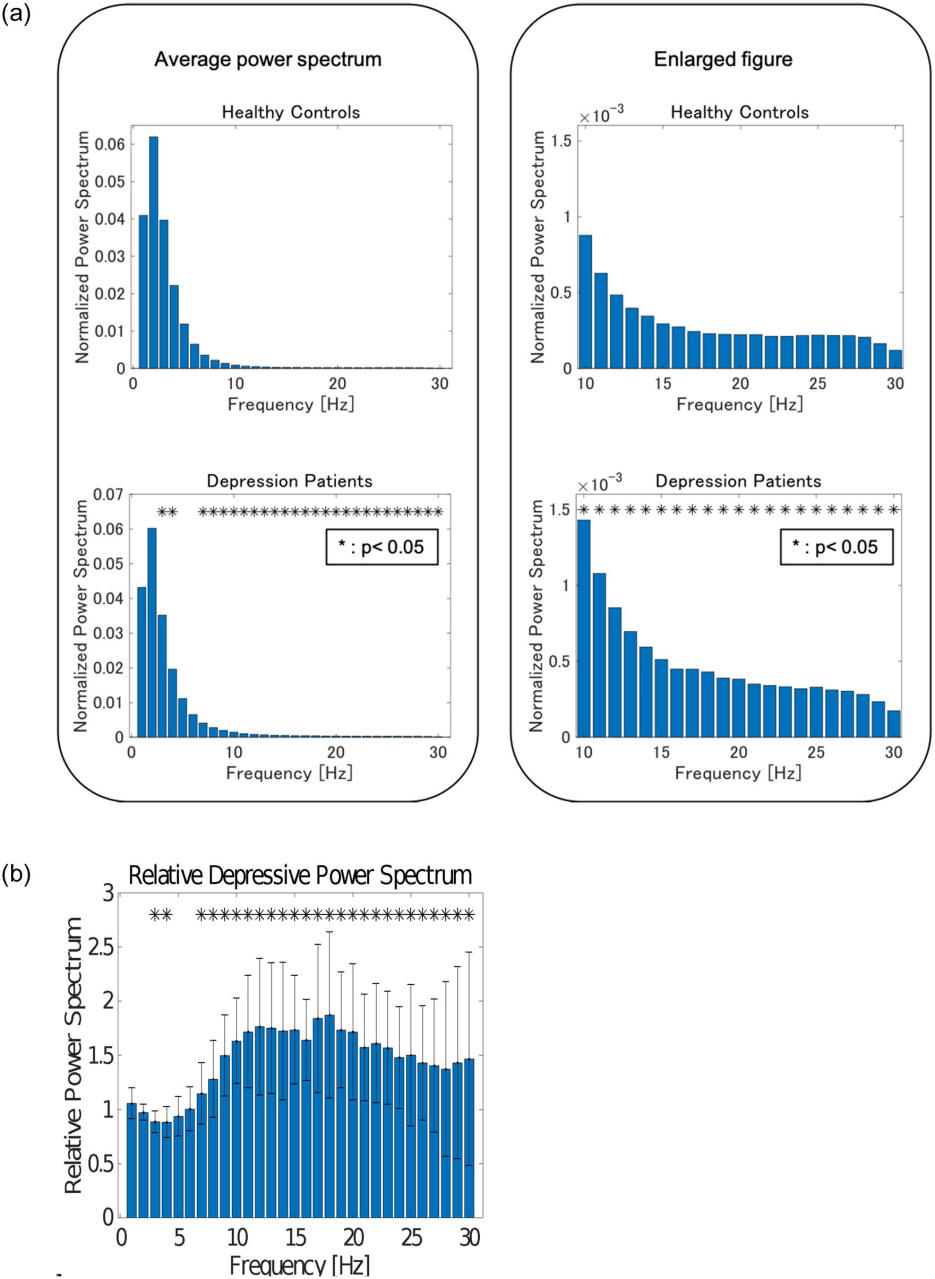
(a). The average normalized power spectrum of depression patients and healthy controls. Significant differences were observed at 3 Hz, 4 Hz, and over 10 Hz. (b). Relative power when healthy controls are set to 1. Significant differences were observed at 3 Hz, 4 Hz, and over 10 Hz.

The average normalized power spectra of patients with depression and healthy controls are shown in Fig 1(a) and 1(b). At 3 Hz, 4 Hz, and 10 Hz and over, power spectra of patients with depression were significantly larger compared to those of healthy individuals.

### 3.3 Comparison of healthy controls and patients with depression grouped by severity level

**Fig 2:**
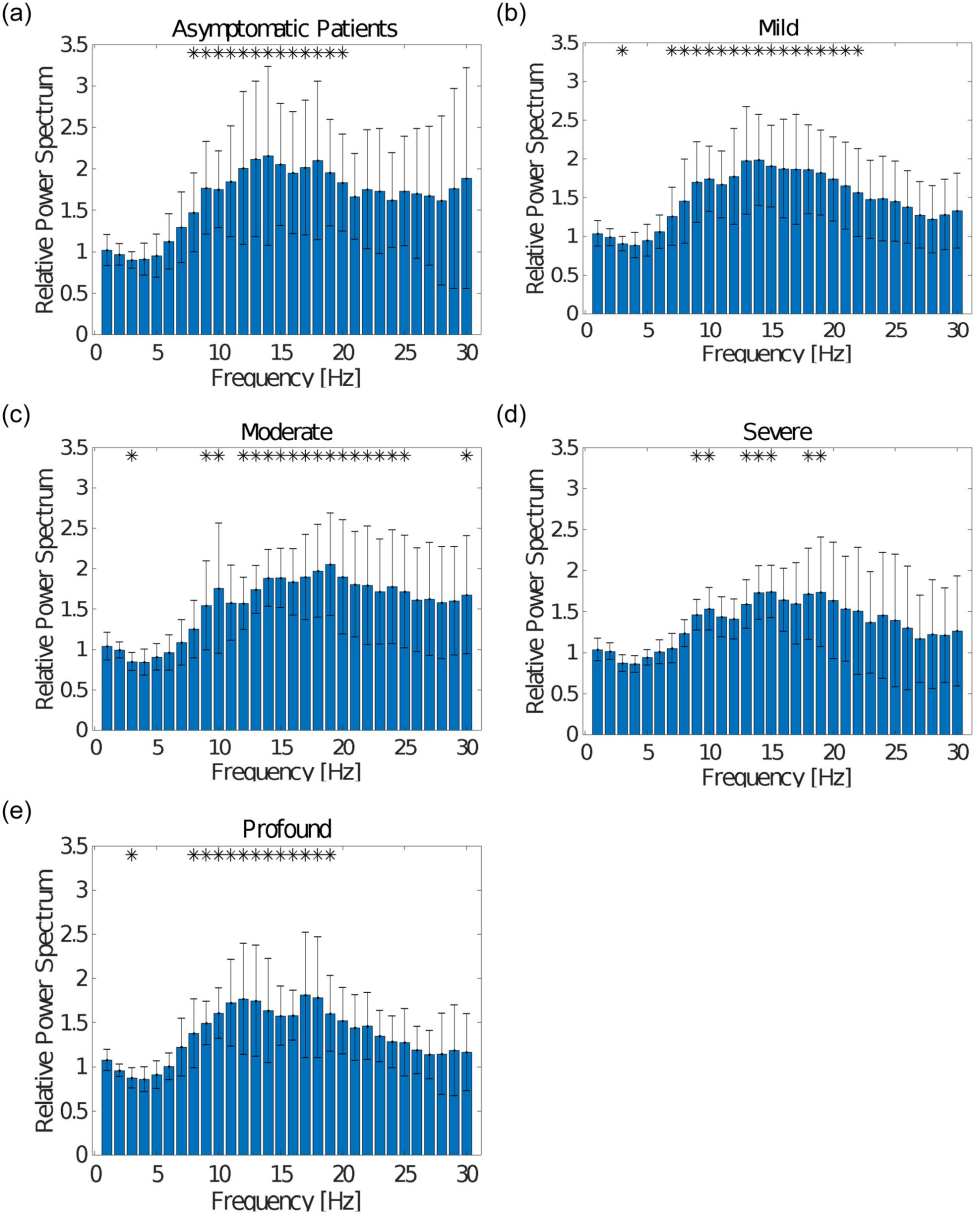
(a) EEG data for 13 patients in the Asymptomatic group (HAMD score 0–7) as compared to healthy controls. Significant differences were observed from 8 Hz to 19 Hz. (b) EEG data for 17 patients in the Mild group (HAMD score 8–13) as compared to healthy controls. Significant differences were observed at 3 Hz and from 7 Hz to 22 Hz. (c) EEG data for 11 patients in the Moderate group (HAMD score 14–18) as compared to healthy controls. Significant differences were observed at 3 Hz, 9 Hz, 10 Hz, and 30 Hz, and from 12 Hz to 24 Hz. (d) EEG data for 8 patients in the Severe group (HAMD score 19–22) as compared to healthy controls. Significant differences were observed at 9 Hz, 10 Hz, 18 Hz, 19 Hz, and 30 Hz, and from 13 Hz to 15 Hz. (e) EEG data for 10 patients in the Profound group (HAMD score 23≤) as compared to healthy controls. Significant differences were observed at 3 Hz, and from 8 Hz to 19 Hz.

The number of data sets of patients with depression in each severity level group was as follows: Mild (HAMD score 8–13): 17; Moderate (HAMD score 14–18): 11; Severe (HAMD score 19–22): 8; and Profound (HAMD score 23≤): 10. In addition, 13 patients’ data sets with normal scores (HAMD score 0–7) were placed in an Asymptomatic group. Fig 2(a)–2(e) show EEG comparisons between healthy controls and patients with Asymptomatic, Mild, Moderate, Severe, and Profound severity. Significant differences were observed in the range of 8 Hz and 19 Hz between Asymptomatic group and healthy individuals. Significant differences were observed at 3 Hz and from 7 Hz to 22 Hz between Mild group and healthy individuals. Similarly, significant differences were observed at 3 Hz, 9 Hz, 10 Hz, from 12 Hz to 24 Hz, and 30 Hz between Moderate group and healthy individuals. Significant differences were also observed at 9 Hz, 10 Hz, from 13 Hz to 15 Hz, 18 Hz, 19 Hz, and 30 Hz between Severe group and healthy individuals. Finally, significant differences were observed at 3 Hz and from 8 Hz to 19 Hz between Profound group and healthy individuals.

### 3.4 Impact of medication on EEG

**Fig 3:**
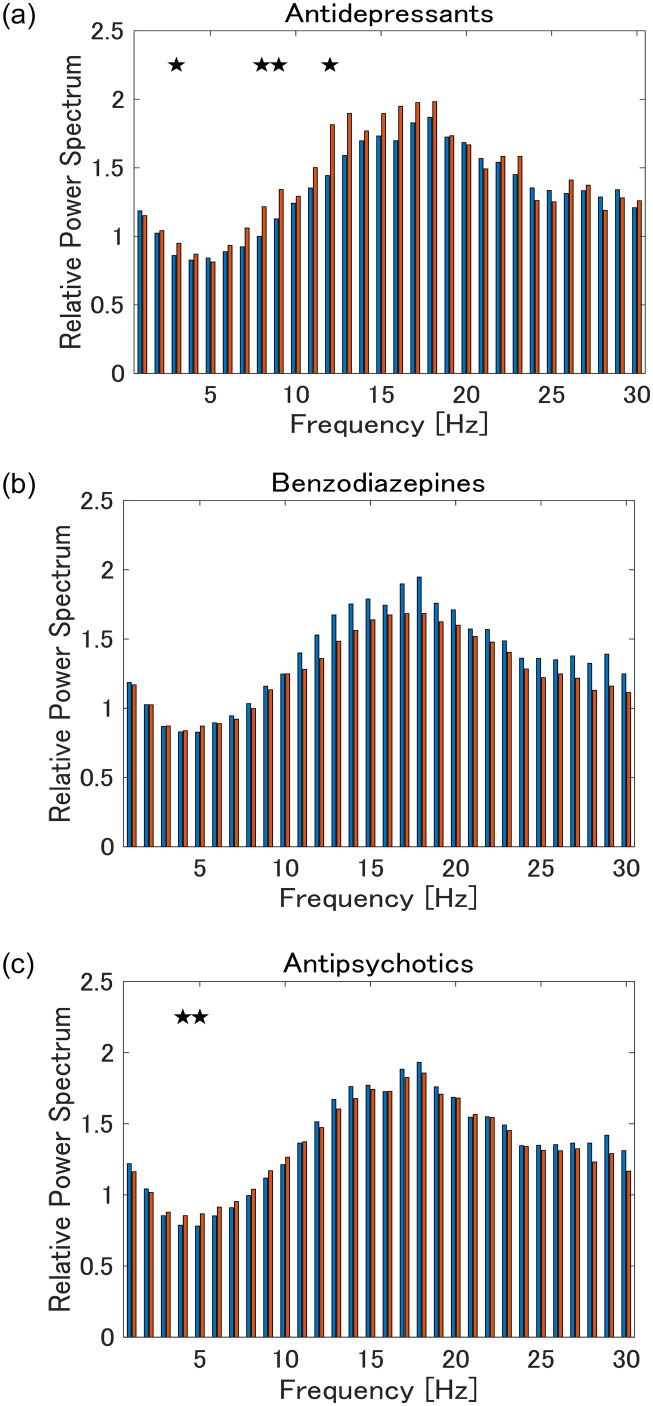
Comparisons of patient groups taking (a) antidepressants, (b) benzodiazepines, and (c) antipsychotics, respectively, and the group not taking medication (Blue: patients taking each medication in their treatment; Red: comparison of patients who did not take the medication). Stars indicate frequencies with significant differences at a significance level of 5% (Also see S3–S5 Figs).

The differences between each of the groups using antidepressants, benzodiazepines, and antipsychotics, and the group not using them, are shown in Fig 3(a)–3(c). EEG differences between patients taking each medication and those not taking them were observed at some frequencies for some drugs. When comparing non-medicated patients and healthy controls, significant power spectra differences were still found between the two groups at multiple frequencies that were similar to those in which we found significant differences between medicated patients and healthy controls (S3–S5 Figs).

## 4 Discussion

In this study, we were able to collect EEG recordings while patients were engaged in conversation by using noise reduction technology. EEG differences between healthy individuals and patients with depression were found in many frequencies of the α and β regions, and there were also EEG differences found based on severity of patients with depression. Furthermore, the results suggested that illness has a greater effect than treatment. The majority of previous research on this topic has focused on resting-state EEG, and have mainly targeted α-waves [20]. However, because we used noise reduction technology to study EEG β-range characteristics while participants engaged in conversation, we were able to observe differences between patients with depression and healthy controls in wider frequency regions. To the best of our knowledge, this is the first study to demonstrate the possibility of differentiating patients with depression and healthy controls through the use of EEG during conversation. We believe this study shows the future potential for this method to be used in diagnosing depression and/or evaluating symptom severity.

There have long been studies done on EEG characteristics of patients with depression, and most of these have been done in quiet, shielded room-type environments using multipolar EEG [5, 6, 21]. This method is time consuming, places a large burden on the participants, and only allows for EEG recordings done in a resting state. Moreover, in order to collect data from the multiple poles, a large amount of statistical verification is required (number of poles * frequencies or frequency bands), and performing multiple comparison correction lowers the statistical power of the study. In comparison, while the single-channel EEG used in this study only measures the frontal lobe, there is very little burden placed on the participants, and recordings are possible even during an active state. Additionally, as stated above, our method is beneficial in terms of statistical comparison in that it does not require consideration of multiple comparisons.

Previous studies have reported a variety of results regarding the differences in EEG between patients with depression and healthy controls. For example, Tesler et al. focused on EEG recordings made during sleep. They showed that patients with depression have a higher spectrum in the slow band than healthy people [3]. While it is interesting to observe the EEG characteristics of patients with depression during sleep, the requirements of this method (having the patient sleep) may be too cumbersome for application in clinical practice.

Towers et al., Sutton et al. and Allen et al. reported that asymmetrical frontal lobe EEG data are connected to depression [22–24]. In particular, a relative decline in activity in the left frontal lobe during resting-state has been found in depression patients. While experimental methods have differed across a number of studies, it is significant that they have all shown a consistent result [25–29]. However, these results are not always highly reproducible, and there are some who have pointed out that anxiety symptoms may affect results [28, 30]. It should be noted that all the aforementioned studies used, at minimum, tripolar electrodes, including the reference electrode.

Additionally, previous research has demonstrated that the slow wave power spectrum is high in patients with depression after performing Fourier transform on the EEG data [3]. Furthermore, there are also reports that in patients with MDD, there are strong slow wave emissions, as well as strong frontal lobe β wave emissions [6]. Also, Pizzagalli et al. found that there were changes in β region activity in the right frontal cortex and upper frontal area of patients with depression [4]. They also stated that this effect was even more pronounced in patients with severe melancholy, and right frontal cortex activity had a strong positive correlation with anxiety.

There are also studies that have identified relationships between treatment response and EEG. Towers et al. reported that occipital lobe α power spectra were different between patients who responded to selective serotonin reuptake inhibitor (SSRI) treatment and those who did not [22]. Additional studies have reported similar findings, which makes these results compelling [31].

In our study, where the EEG data of patients with depression and healthy controls were recorded during conversation and compared, the power spectra of the patients’ EEG were significantly different from those of the healthy controls, particularly in the high frequency region. On the other hand, it became clear after analysis that there was no difference in the spectrum in the slow wave band for healthy controls and patients. Our discovery is similar to those of the above-mentioned studies by Grin-Yatsenko et al. [6, 32] and Pizzagalli et al. [4] Grin-Yatsenko et al. found a parietal increase in β waves, and we were able to confirm an increase in β waves in the frontal lobe as well [32]. Also, Pizzagalli et al. found an acceleration in right frontal cortex and upper frontal area activity, which does not conflict with our findings [4].

We found differences in β waves in our EEG recordings, but we must ask what physiological meaning this represents. Regarding the relationship between stress and EEG measured subjective stress, urinary cortisol, and EEG in six healthy persons under high-stress conditions simulating a spacecraft environment. They reported that higher cortisol levels correlated with enhanced EEG β-components [33].

Weinstein et al. considered that patients with depression did not respond properly to negative feedback, and responded excessively to the stress response [34]. They stated that if patients with depression respond excessively to the stress response, this could cause β to increase.

Our study found that as symptom severity changes, differences in the frequencies and/or power spectrum appeared, but without a consistent and/or proportional pattern. It is possible that people with severe symptoms usually take more medication, which may influence the EEG results. Studies that target more non-medicated patients, or ones that have larger sample size, are needed.

In regard to the effects of psychotropic drugs on EEG, current knowledge depends on the specific drug; some still lack the evidence needed to determine their effects, and in many cases, results are not consistent [35, 36]. However, studies that reported comparatively consistent results listed the following effects. For antidepressants, tricyclic types weakened the α and enhanced the β. SSRI enhanced the slow wave and β, and weakened the α. Noradrenergic and specific serotonergic antidepressants (NaSSA) enhanced the slow wave and α [37–39]. For antipsychotics, multi-acting receptor targeted antipsychotics (MARTA) enhanced the slow wave and β [37–39], and weakened the α. Benzodiazepines weakened the slow wave and α, and enhanced the β [36–38, 40, 41].

Patients included in our study sample were using the above-mentioned medications, and those medications may have influenced our results. After dividing patients into subgroups based on whether or not they were taking antidepressants, benzodiazepines, or antipsychotics, and comparing their EEG data, we found differences in several frequency powers. However, those differences were few in number, and even in the non-medicated group, there were notable differences compared with the healthy controls, so we believe there was some direct effect from the illness on patients.

This study has the following limitations. First, our sample size was small, which may have caused Type II error. For example, we did not find any kind of EEG characteristics that were proportional to depression severity, but a larger number of participants may have allowed us to discover such characteristics. It is also possible that, due to the small sample size, there were incidental findings made. Second, patients’ demographic characteristics were not controlled, and their educational background and age were not uniform. Third, most patients were taking prescribed medication, and it is possible that these drugs interfered with the EEG results. However, as stated above, because we found differences between the non-medicated group and healthy controls as well, we believe that there was a direct disease effect on EEG results to some extent.

We adopted only FP1 location in 10–20 system in this study, but it was not necessary the optimal location and further study to verify it is needed.

Finally, gamma involvement has already been recognized in various diseases, but it was not examined in this paper.

## 5 Conclusions

By acquiring EEG data during conversation using noise reduction technology, differences in EEG between healthy individuals and patients with depression, as well as differences in depression symptom severity, were observed. This investigation should be done with a larger sample size to gauge reproducibility, but the methods used in this study to detect depression are simple to implement in clinical settings, and could be useful in psychiatric clinical practice, as biomarkers in the psychiatric field are scarce.

## Supporting information

S1 Fig International 10–20 system. (DOCX)

S2 Fig EEG measuring device ©Dentsu science JAM. (DOCX)

S3 Fig (a) (b): The differences between groups using antidepressants. (DOCX)

S4 Fig (a) (b): The differences between groups using benzodiazepines. (DOCX)

S5 Fig (a) (b): The differences between groups using antipsychotics. (DOCX)

S1 Text Pseudocode of the experimental procedure. (DOCX)

## References

[1] Lemere F. THE SIGNIFICANCE OF INDIVIDUAL DIFFERENCES IN THE BERGER RHYTHM. *Brain*. 1936;59(3):366–75.

[2] Stewart JL, Allen JJ. Resting frontal brain asymmetry is linked to future depressive symptoms in women. *Biological Psychology*. 2018 Jul;136:161–7. doi: 10.1016/j.biopsycho.2018.06.004 29920297 PMC6084483

[3] Tesler N, Gerstenberg M, Franscini M, Jenni OG, Walitza S, Huber R. Increased frontal sleep slow wave activity in adolescents with major depression. *NeuroImage*: *Clinical*. 2016;10:250–6. doi: 10.1016/j.nicl.2015.10.014 26870661 PMC4712324

[4] Pizzagalli DA, Nitschke JB, Oakes TR, Hendrick AM, Horras KA, Larson CL, et al. Brain electrical tomography in depression: the importance of symptom severity, anxiety, and melancholic features. *Biological Psychiatry*. 2002 Jul;52(2):73–85. doi: 10.1016/s0006-3223(02)01313-6 12113998

[5] Roh S-C, Kim JS, Kim S, et al. Frontal Alpha Asymmetry Moderated by Suicidal Ideation in Patients with Major Depressive Disorder: A Comparison with Healthy Individuals. *Clin Psychopharmacol Neurosci* 2020; 18: 58–66. doi: 10.9758/cpn.2020.18.1.58 31958906 PMC7006982

[6] Grin-Yatsenko VA, Baas I, Ponomarev VA, Kropotov JD. Independent component approach to the analysis of EEG recordings at early stages of depressive disorders. *Clinical Neurophysiology*. 2010 Mar;121(3):281–9. doi: 10.1016/j.clinph.2009.11.015 20006545

[7] Hamilton M. A rating scale for depression. *Journal of Neurology*, *Neurosurgery*, *and Psychiatry*. 1960; 23 (1): 56–62. doi: 10.1136/jnnp.23.1.56 . 14399272 PMC495331

[8] Shen J, Zhao S, Yao Y, Wang Y, Feng L. A novel depression detection method based on pervasive EEG and EEG splitting criterion. *2017 IEEE International Conference on Bioinformatics and Biomedicine (BIBM)*. IEEE; 2017.

[9] Nakamura R, Mitsukura Y. Feature analysis of electroencephalography in patients with depression. *2018 IEEE Life Sciences Conference (LSC)*. IEEE; 2018.

[10] Ratti E, Waninger Waninger S, Berka C, et al. Comparison of Medical and Consumer Wireless EEG Systems for Use in Clinical Trials. *Front Hum Neurosci*; 11. Epub ahead of print August 3, 2017. doi: 10.3389/fnhum.2017.00398 28824402 PMC5540902

[11] Kandel E, Schwartz JH Jessel T, Siegelbaum S. *Principles of Neural Science*. 5th ed. New York: McGraw-Hill Professional, 2012.

[12] Ogino M, Mitsukura Y. Portable Drowsiness Detection through Use of a Prefrontal Single-Channel Electroencephalogram. *Sensors*. 2018 Dec 18;18(12):4477. doi: 10.3390/s18124477 30567347 PMC6308812

[13] Mitsukura Y, Sumali B, Tazawa Y, Kishimoto T, Mimura M. Simple Stress Quantitative Evalution for Healthcare Using Daily KANSEI Detection with EEG Device-Relation between Stress and Healthcare. *Mod*. *Environ*. *Sci*. *Eng*. 2019;5:345–350.

[14] KANOGA S, MITSUKURA Y. A Study of Pattern Recognition in Children Using Single-Channel Electroencephalogram for Specialized Electroencephalographic Devices. *Electron Comm Jpn* 2017; 100: 43–53.

[15] Prasanna J, Subathra MSP, Mohammed MA, Damaševičius R, Sairamya NJ, George ST. Automated epileptic seizure detection in pediatric subjects of CHB-MIT EEG database-A survey. *J Pers Med*. 2021;11: 1028. doi: 10.3390/jpm11101028 34683169 PMC8537151

[16] Iaccarino HF, Singer AC, Martorell AJ, Rudenko A, Gao F, Gillingham TZ, et al. Gamma frequency entrainment attenuates amyloid load and modifies microglia. *Nature*. 2016;540: 230–235. doi: 10.1038/nature20587 27929004 PMC5656389

[17] van Deursen JA, Vuurman EFPM, Verhey FRJ, van Kranen-Mastenbroek VHJM, Riedel WJ. Increased EEG gamma band activity in Alzheimer’s disease and mild cognitive impairment. *J Neural Transm (Vienna)*. 2008;115: 1301–1311. doi: 10.1007/s00702-008-0083-y 18607528 PMC2525849

[18] Cea-Cañas B, Díez Á, Lubeiro A, Iglesias M, Capella C, Rodríguez-Lorenzana A, et al. Altered gamma band noise power in schizophrenia and bipolar patients during a cognitive task. *Eur J Psychiatry*. 2021;35: 129–139. doi: 10.1016/j.ejpsy.2020.11.003

[19] Pal A, Pegwal N, Behari M, Sharma R. High delta and gamma EEG power in resting state characterise dementia in Parkinson’s patients. *Biomarkers in Neuropsychiatry*. 2020;3: 100027. doi: 10.1016/j.bionps.2020.100027

[20] Olbrich S, van Dinteren R, Arns M. Personalized Medicine: Review and Perspectives of Promising Baseline EEG Biomarkers in Major Depressive Disorder and Attention Deficit Hyperactivity Disorder. *Neuropsychobiology* 2015; 72: 229–240. doi: 10.1159/000437435 26901357

[21] Mahato S, Paul S. Classification of Depression Patients and Normal Subjects Based on Electroencephalogram (EEG) Signal Using Alpha Power and Theta Asymmetry. *J Med Syst*; 44. Epub ahead of print December 13, 2019. doi: 10.1007/s10916-019-1486-z 31834531

[22] Towers DN, Allen JJB. A better estimate of the internal consistency reliability of frontal EEG asymmetry scores. *Psychophysiology* 2009; 46: 132–142. doi: 10.1111/j.1469-8986.2008.00759.x 19055503 PMC2743447

[23] Sutton SK, Davidson RJ. Prefrontal Brain Asymmetry: A Biological Substrate of the Behavioral Approach and Inhibition Systems. *Psychol Sci* 1997; 8: 204–210.

[24] Allen JJ, Reznik SJ. Frontal EEG asymmetry as a promising marker of depression vulnerability: summary and methodological considerations. *Current Opinion in Psychology* 2015; 4: 93–97. doi: 10.1016/j.copsyc.2014.12.017 26462291 PMC4599354

[25] Schaffer CE, Davidson RJ, Saron C. Frontal and parietal electroencephalogram asymmetry in depressed and nondepressed subjects. *Biol Psychiatry*. 1983 Jul;18(7):753–62. 6615936

[26] Gotlib IH. EEG Alpha Asymmetry, Depression, and Cognitive Functioning. *Cognition & Emotion* 1998; 12: 449–478.

[27] Henriques JB, Davidson RJ. Left frontal hypoactivation in depression. *Journal of Abnormal Psychology* 1991; 100: 535–545. doi: 10.1037//0021-843x.100.4.535 1757667

[28] Thibodeau R, Jorgensen RS, Kim S. Depression, anxiety, and resting frontal EEG asymmetry: A meta-analytic review. *Journal of Abnormal Psychology* 2006; 115: 715–729. doi: 10.1037/0021-843X.115.4.715 17100529

[29] Davidson RJ. Cerebral asymmetry and emotion: Conceptual and methodological conundrums. *Cognition & Emotion* 1993; 7: 115–138.

[30] Mathersul D, Williams LM, Hopkinson PJ, et al. Investigating models of affect: Relationships among EEG alpha asymmetry, depression, and anxiety. *Emotion* 2008; 8: 560–572. doi: 10.1037/a0012811 18729586

[31] Baskaran A, Milev R, McIntyre RS. The neurobiology of the EEG biomarker as a predictor of treatment response in depression. *Neuropharmacology* 2012; 63: 507–513. doi: 10.1016/j.neuropharm.2012.04.021 22569197

[32] Grin-Yatsenko VA, Baas I, Ponomarev VA, et al. EEG Power Spectra at Early Stages of Depressive Disorders. *Journal of Clinical Neurophysiology* 2009; 26: 401–406. doi: 10.1097/WNP.0b013e3181c298fe 19952564

[33] Gemignani A, Piarulli A, Menicucci D, et al. How stressful are 105days of isolation? Sleep EEG patterns and tonic cortisol in healthy volunteers simulating manned flight to Mars. *International Journal of Psychophysiology* 2014; 93: 211–219. doi: 10.1016/j.ijpsycho.2014.04.008 24793641

[34] Weinstein AA, Deuster PA, Francis JL, et al. Neurohormonal and inflammatory hyper-responsiveness to acute mental stress in depression. *Biological Psychology* 2010; 84: 228–234. doi: 10.1016/j.biopsycho.2010.01.016 20117167 PMC2875322

[35] Saletu B, Anderer P, Saletu-Zyhlarz GM. EEG Topography and Tomography (LORETA) in the Classification and Evaluation of the Pharmacodynamics of Psychotropic Drugs. *Clin EEG Neurosci* 2006; 37: 66–80. doi: 10.1177/155005940603700205 16733939

[36] Aiyer R, Novakovic V, Barkin RL. A systematic review on the impact of psychotropic drugs on electroencephalogram waveforms in psychiatry. *Postgraduate Medicine* 2016; 128:7, 656–664. doi: 10.1080/00325481.2016.1218261 27467441

[37] Schmid DA, Wichniak A, Uhr M, et al. Changes of Sleep Architecture, Spectral Composition of Sleep EEG, the Nocturnal Secretion of Cortisol, ACTH, GH, Prolactin, Melatonin, Ghrelin, and Leptin, and the DEX-CRH Test in Depressed Patients during Treatment with Mirtazapine. *Neuropsychopharmacol* 2005; 31: 832–844. 10.1038/sj.npp.1300923 16237393

[38] Lozano-Soldevilla D, ter Huurne N, Cools R, et al. GABAergic Modulation of Visual Gamma and Alpha Oscillations and Its Consequences for Working Memory Performance. *Current Biology* 2014; 24: 2878–2887 doi: 10.1016/j.cub.2014.10.017 25454585

[39] Rohit A, Vladan N, Robert LB, *Postgraduate Medicine*, 128:7, 656–664 doi: 10.1080/00325481.2016.1218261 27467441

[40] Schittecatte M, Dumont F, Machowski R, et al. Mirtazapine, but not fluvoxamine, normalizes the blunted REM sleep response to clonidine in depressed patients: implications for subsensitivity of alpha2-adrenergic receptors in depression. *Psychiatry Research* 2002; 109: 1–8. doi: 10.1016/s0165-1781(01)00362-6 11850045

[41] Tislerova B, Brunovsky M, Horacek J, et al. LORETA Functional Imaging in Antipsychotic-Naive and Olanzapine-, Clozapine- and Risperidone-Treated Patients with Schizophrenia. *Neuropsychobiology* 2008; 58: 1–10. doi: 10.1159/000154474 18781085

