# Supplementary material for: Characteristics of single-channel electroencephalogram in depression during conversation with noise reduction technology
Source: PLoS One. 2022 Apr 13;17(4):e0266518. doi: 10.1371/journal.pone.0266518 (PMC9007370; doi:10.1371/journal.pone.0266518)
Supplement: S1 Fig — (DOCX) [file pone.0266518.s001.docx]

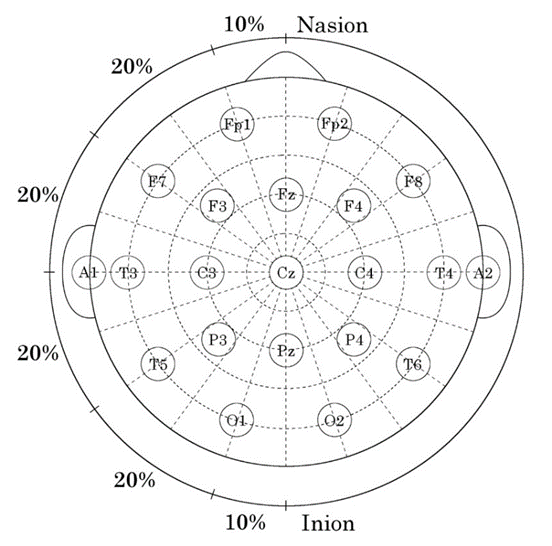


**S1 Fig. International 10-20 system.** (The center of nausal, occipital nodules and left and right auricles are defined as Cz. From Cz, toward the lower part of the brain and on the circumference centered on Cz, the areas are divided into 20%, 20%, 10% respectively).
