# Supplementary figures and images for: Characteristics of single-channel electroencephalogram in depression during conversation with noise reduction technology
Source: PLoS One. 2022 Apr 13;17(4):e0266518. doi: 10.1371/journal.pone.0266518 (PMC9007370; doi:10.1371/journal.pone.0266518)

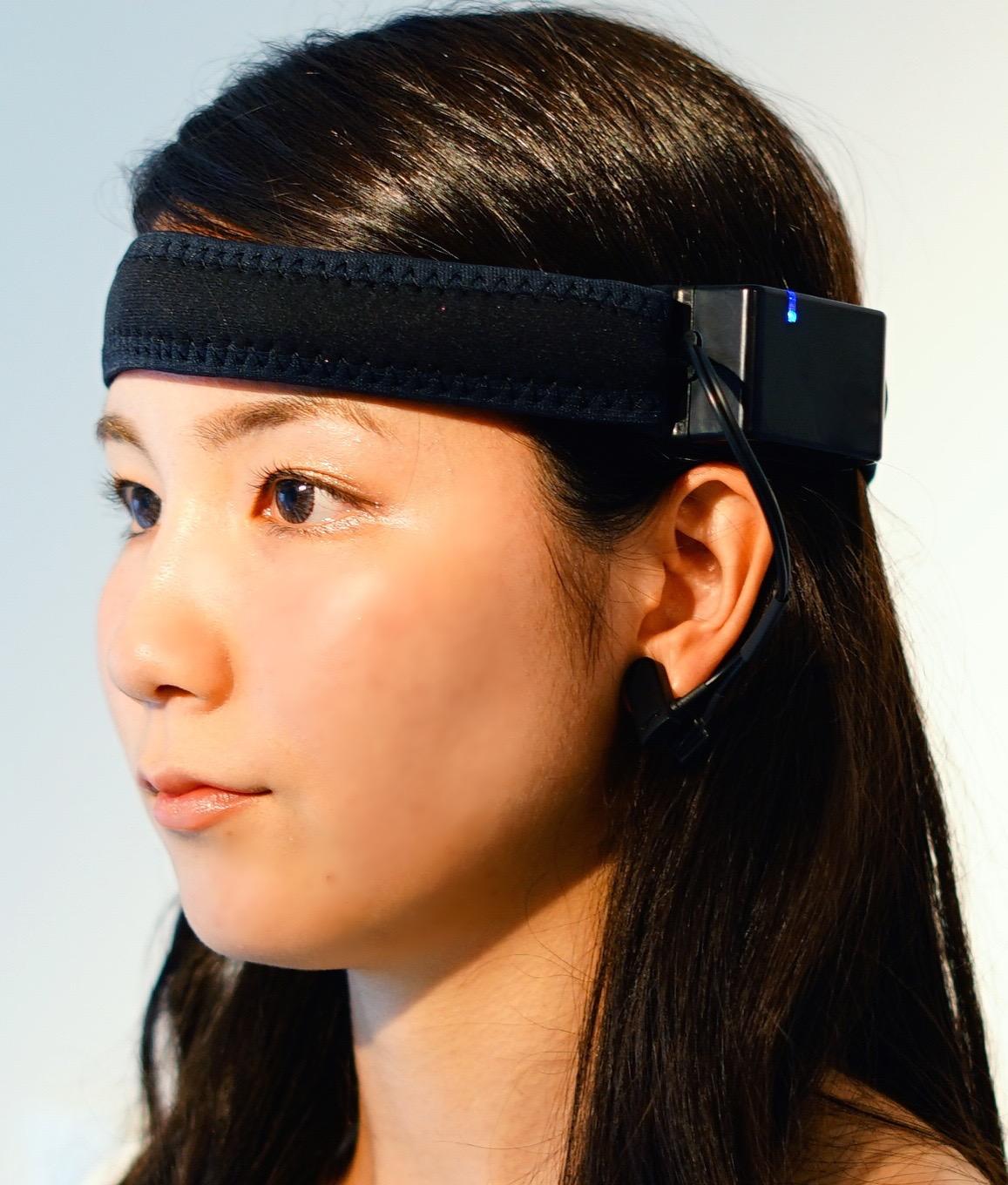

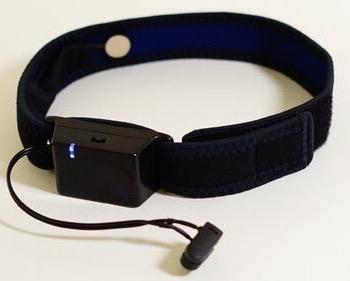


**S2 Fig. EEG measuring device ©Dentsu science JAM.** It has an electrode and an ear clip.

Supplement: S2 Fig — (DOCX) [file pone.0266518.s002.docx]
