# Supplementary material for: Characteristics of single-channel electroencephalogram in depression during conversation with noise reduction technology
Source: PLoS One. 2022 Apr 13;17(4):e0266518. doi: 10.1371/journal.pone.0266518 (PMC9007370; doi:10.1371/journal.pone.0266518)
Supplement: S3 Fig — (DOCX) [file pone.0266518.s003.docx]

(a) Frequencies at which there was a significant difference in the power spectrum between patients not taking antidepressants (red) and healthy controls are marked with a star.

(b) Frequencies at which there was a significant difference in the power spectrum between patients taking antidepressants (blue) and healthy controls are marked with a star.

**S3 Fig. The differences between groups using antidepressants.**

Differences between the group using antidepressants and the group not using them when compared with healthy controls, with healthy controls assigned a value of 1. (Blue: shows EEG data for patients taking antidepressants with healthy controls’ value set at 1. Red: shows EEG data for patients not taking antidepressants with healthy controls’ value set at 1.)

(a) Frequencies at which there was a significant difference between patients not taking antidepressants and healthy controls are marked.

(b) Frequencies at which there was a significant difference between patients taking antidepressants and healthy controls are marked.
