# Supplementary material for: Characteristics of single-channel electroencephalogram in depression during conversation with noise reduction technology
Source: PLoS One. 2022 Apr 13;17(4):e0266518. doi: 10.1371/journal.pone.0266518 (PMC9007370; doi:10.1371/journal.pone.0266518)
Supplement: S1 Text — (DOCX) [file pone.0266518.s006.docx]

**S1 Text. Pseudocode of the experimental procedure.**

define EEG_SR 512

main(){

init Study_Database as VECTOR

Subjects = recruitment( Healthy_Volunteers || Patients );

foreach Patient in Subjects{

Data = acquisition( Patient )

Study_Database.insert(Data)

}

Results = analysis( Study_Database )

print( Results )

}

Sample = recruitment( Population ){

init Sample as VECTOR

foreach Patient in Population{

if ( inclusion_criteria(Patient) && !exclusion_criteria(Patient) ){

Bool_consent = ask_consent()

if Bool_consent:

Sample.insert(Patient)

}

}

return Sample

}

Data = acquisition( Patient ){

init Data as STRUCT

init Doctor as PERSON

init EEG_Device as SENSOR_HARDWARE

init EEG_Device_App as SENSOR_SOFTWARE

while(true){

Data.Num_experiment = Patient.get_num_experiment()

if (Data.Num_experiment == 0){

Data.Sociodemographic = Patient.get_sociodemographic()

}

Data.Num_experiment++

Patient.set_num_experiment(Data.Num_experiment)

Patient.equip(EEG_Device)

Doctor.equip(EEG_Device_App)

Doctor.instruct(Patient, Message_close_eye)

Doctor.control(EEG_Device_App,start_calibrate)

waitfor(EEG_Device_App.finish_calibration() == true)

Doctor.control(EEG_Device_App,end_calibrate)

Doctor.instruct(Patient, Message_open_eye)

waitfor(Patient.is_relaxed() == true)

Doctor.control(EEG_Device_App,start_EEG_recording)

HAMD_Score = Doctor.interview(HAMD, Patient) //approx 40 mins

Doctor.control(EEG_Device_App,stop_EEG_recording)

Patient.unequip(EEG_Device)

Next_appointment = Doctor.ask(Patient, Message_next_appointment)

if(Next_appointment != NULL){

Doctor.Calendar.insert(Next_appointment)

Patient.Calendar.insert(Next_appointment)

}

Data.EEG.insert(Doctor.control(EEG_Device_App,export_rawdata)) //EEG data is a 1D array

Data.HAMD.insert(HAMD_Score)

Doctor.unequip(EEG_Device_App)

if(Next_appointment != NULL && Data.Num_experiment < 3){

sleepuntil(Next_appointment)

}else{

return Data

}

}

}

Results = analysis( Study_Database ){

init PSD_Results as VECTOR

init Demographic_Results as VECTOR

init Vector_HAMD as VECTOR

init Results as STRUCT

//feature extraction

foreach Data in Study_Database{

Index_MaxHAMD = findmax(Data.HAMD)

EEG_Signal = Data.EEG[ Index_MaxHAMD ] //in this pseudocode, array indices start from 1, not 0

EEG_Pre1 = bandpass_filter(EEG_Signal, [1,30], EEG_SR) //512 is the EEG's SR (sampling ratio)

EEG_Pre2 = conventional_noiseremoval(EEG_Pre1)

EEG_Normalized = array_elementwise_division( array_subtraction(EEG_Pre2, mean(EEG_Pre2) ), std_dev(EEG_Pre2) )

EEG_FFT = fourier_transform( EEG_Normalized )

PSD = array_elementwise_division( array_elementwise_multiplication(EEG_FFT,EEG_FFT), length(EEG_Normalized) )

PSD = PSD[1:EEG_SR/2+1]

PSD[2:length(PSD)-1] = array_elementwise_multiplication(PSD[2:length(PSD)-1] , 2)

PSD_Results.insert( PSD )

Demographic_Results.insert( Data.Sociodemographic )

Vector_HAMD.insert( Data.HAMD[ Index_MaxHAMD ] )

}

Matrix_PSD_Results = vector2matrix( PSD_Results ) //row = samples, col = frequencies [0, SR/2]

Matrix_Demographic_Results = vector2matrix( Demographic_Results ) //row = samples, col = variables (age, sex, educational years, etc.)

Vector_Is_Depression = Vector_HAMD >= 8

Results.Demographic_Normality = check_normality(Matrix_Demographic_Results)

Results.PSD_Normality = check_normality(Matrix_PSD_Results)

Results.TTest2 = ttest2( Matrix_PSD_Results[Vector_Is_Depression], Matrix_PSD_Results[!Vector_Is_Depression] )

return Results

}

A = array_subtraction(A, B){

if(length(B) == 1){

for Index_A = 1:length(A){

A[Index_A] = A[Index_A] - B

}

}else{

for Index_A = 1:length(A){

A[Index_A] = A[Index_A] - B[Index_A]

}

}

return A

}

A = array_elementwise_division(A, B){

if(length(B) == 1){

for Index_A = 1:length(A){

A[Index_A] = A[Index_A] / B

}

}else{

for Index_A = 1:length(A){

A[Index_A] = A[Index_A] / B[Index_A]

}

}

return A

}

A = array_elementwise_multiplication(A, B){

if(length(B) == 1){

for Index_A = 1:length(A){

A[Index_A] = A[Index_A] * B

}

}else{

for Index_A = 1:length(A){

A[Index_A] = A[Index_A] * B[Index_A]

}

}

return A

}
